# Lactosaminated mesoporous silica nanoparticles for asialoglycoprotein receptor targeted anticancer drug delivery

**DOI:** 10.1186/s12951-015-0068-6

**Published:** 2015-02-03

**Authors:** Guilan Quan, Xin Pan, Zhouhua Wang, Qiaoli Wu, Ge Li, Linghui Dian, Bao Chen, Chuanbin Wu

**Affiliations:** School of Pharmaceutical Sciences, Sun Yat-Sen University, Guangzhou, 510006 People’s Republic of China; Guangzhou Neworld Pharmaceutical Ltd. Co., Guangzhou, 510006 People’s Republic of China; School of Pharmaceutical Sciences, Guangdong Medical College, Dongguan, 523808 People’s Republic of China

**Keywords:** Mesoporous silica nanoparticles, Lactose, Asialoglycoprotein receptor, Docetaxel

## Abstract

**Background:**

Mesoporous silica nanoparticles (MSNs) have several attractive properties as a drug delivery system, such as ordered porous structure, large surface area, controllable particle size as well as interior and exterior dual-functional surfaces. The purpose of this study was to develop novel lactosaminated mesoporous silica nanoparticles (Lac-MSNs) for asialoglycoprotein receptor (ASGPR) targeted anticancer drug delivery.

**Results:**

Lac-MSNs with an average diameter of approximately 100 nm were prepared by conjugation of lactose with 3-aminopropyl triethoxysilane modified MSNs. Characterization of Lac-MSNs indicated a huge Brunauer-Emmett-Teller (BET) surface area (1012 m^2^/g), highly ordered 2D hexagonal symmetry, an unique mesoporous structure with average pore size of 3.7 nm. The confocal microscopy and flow cytometric analysis illustrated Lac-MSNs were effectively endocytosed by ASGPR-positive hepatoma cell lines, HepG2 and SMMC7721. In contrast, non-selective endocytosis of Lac-MSNs was found in ASGPR-negative NIH 3T3 cells. The cellular uptake study showed the internalization process was energy-consuming and predominated by clathrin-mediated pathway. Model drug docetaxel (DTX) was loaded in the mesopores of Lac-MSNs by wetness impregnation method. *In vitro* cytotoxicity assay showed that DTX transported by Lac-MSNs effectively inhibited the growth of HepG2 and SMMC7721 cells in a time- and concentration- dependent manner.

**Conclusions:**

These results demonstrated that Lac-MSNs could be a promising inorganic carrier system for targeted intracellular anti-cancer drug delivery.

## Background

In recent years, more than ten million people per year worldwide have suffered from cancers, and cancer is one of the deadliest killers to human being [1]. Currently, systemic chemotherapy is the indispensable treatment for malignant tumors. However, many anticancer drugs have severe toxic side effects due to their unspecific actions on normal cells and tissues [2]. Therefore, development of an effective cancer targeting drug delivery system is extremely necessary for improving the drug efficacy to cancer cells, reducing toxic side effects systematically, and prolonging survivals of patients.

With recent advances in nanotechnology research, nanocarries have shown great potential to improve the therapeutic efficacy while minimize the side effects, especially for highly toxic anticancer drugs [3,4]. It is known that the vascular architecture and the lymphatic system in tumors are impaired and may allow the permeation of macromolecules. So, passive targeting of nanocarriers to these abnormal tumors may be partially achieved with the enhanced permeability and retention (EPR) effect [5,6], leaving the surrounding healthy tissues barely touched. It is expected that the application of nanotechnology would be beneficial to millions of cancer patients with more efficient, safe, and affordable treatment.

Though the common organic nanocarriers including polymeric micelles [7], nanocapsules [8], polymer nanoparticles [9], and liposomes [10] have been extensively studied, their physicochemical instability and undesirable drug leakage have severely impeded their further applications. In contrast, inorganic silicate (SiO_2_) carriers possess many advantages, such as great physicochemical and biochemical stabilities, good biocompatibility, and excellent degradability [11]. Recently, silica nanoparticles in the form of Cornell dots (C dots) received FDA’s approval for stage I human clinical trial [12-14], representing an important step towards clinical acceptance of silica-based nanoparticles.

Among silica-based nanomaterials, mesoporous silica nanoparticles (MSNs) have attracted great attention due to their unique properties, including highly regular mesoporous structure, tunable pore size (2–10 nm), huge surface area (>700 m^2^/g), large pore volume (>1 cm^3^/g), excellent endocytotic behavior, and good biocompatibility both *in vitro* and *in vivo* [15-17]. Several chemotherapeutic agents have been successfully delivered by using MSNs as cancer cell-specific delivery vehicles [18-20]. More importantly, the external surface of MSNs can be modified with tumor-recognition molecules to increase the active targetability through the receptor-mediated endocytosis. Several well-known targeting molecules, such as folate [21], mannose [22], hyaluronic acid [23], arginine-glycine-aspartate (RGD) [24], and lactobionic acid [25] have been conjugated to MSNs successfully, resulting in significantly enhanced antitumor efficiency.

Among various targeting ligands, lactose, a glucosyl-galactose disaccharide, shows great promise as a tumor-homing agent, because it has a specific interaction with the asialoglycoprotein receptor (ASGPR) which is a well-characterized molecular target expressed on the cell surface of hepatocytes and hepatomas. ASGPR can actively internalize the bound galactose or galactose-derived complexes via receptor-mediated endocytosis [26,27]. Moreover, due to its low cost, nonimmunogenicity, high stability, and ease for modification, lactose has been recognized as a promising candidate for hepatocellular carcinoma targeting agent. Many researchers have applied lactose to target drug delivery system [28-30]. However, to the best of our knowledge, there is no report on combining lactose with MSNs to construct a drug delivery system for hepatocellular carcinoma targeting.

**Figure 1:**
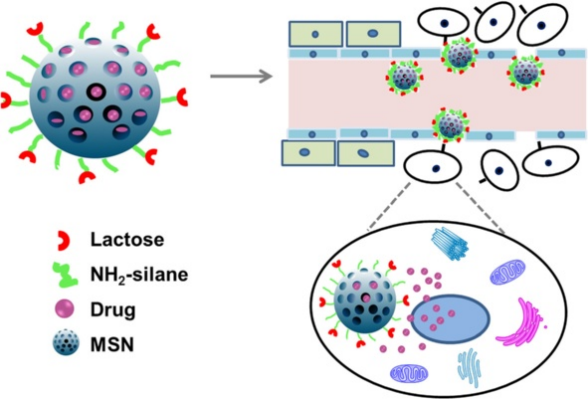
**Schematic diagram of lactosaminated mesoporous silica nanoparticles.**

So, in this study, the targeting property of lactose was integrated with the excellent drug delivery and endocytotic behaviors of MSNs to build a novel drug delivery system, which was expected to possess not only a passive targeting capability via EPR effect but also an active targeting character (Figure 1). Moreover, the internalization mechanism of MSNs by hepatoma cells was investigated to thoroughly understand the efficiency of the lactosaminated MSNs.

## Results and discussion

### Preparation and characterization of MSNs and Lac-MSNs

MSNs were synthesized by the sol–gel method using surfactant as the template. The as-synthesized MSNs prior to removing the template were firstly functionalized with NH_2_-silane on the outer surface, while leaving the inner pores available for drug loading. After conjugation of MSNs with lactose, the template was removed by refluxing the product in acidic ethanol. In addition, fluorescein isothiocyanate (FITC) as a fluorescent probe was encapsulated in the Lac-MSNs through co-condensation in order to monitor the interaction between the nanoparticles and the cells [31].

**Figure 2:**
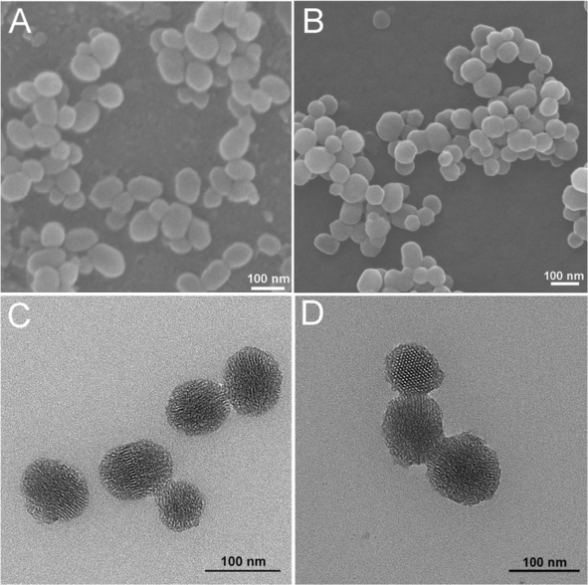
**SEM images of MSNs (A) and Lac-MSNs (B); TEM images of MSNs (C) and Lac-MSNs (D).**

The scanning electron microscopy (SEM) and transmission electron microscopy (TEM) images (Figure 2) showed that both MSNs and Lac-MSNs were roughly spherical in shape and uniform in diameter of approximately 100 nm. The mesoporous structure of MSNs was revealed in details by TEM, as the clearly observed bright and dark domains (Figure 2C and D), corresponding to the pores and the silica walls respectively, confirmed the hexagonal arrays of nanochannels. It is known the particle size of nanoparticles plays an important role on pharmacokinetics. Nanoparticles with particle size smaller than 200 nm can generally increase accumulation of anticancer drug in tumor via EPR effect [5]. Though the particle size of Lac-MSNs was measured as approximately 100 nm based on the TEM images, this only represented the size of inorganic silica core, while the organic NH_2_-silane coating was transparent under TEM observation [32]. Therefore, dynamic light scattering (DLS) was employed to measure the overall size of Lac-MSNs as 170 nm approximately. The difference in particle size obtained from TEM and DLS measurements confirmed the successful deposition of a NH_2_-silane layer on the nanoparticle surface. These silane-layer coated, well-dispersed, small nanoparticles should be favorable for passive tumor targeting and cellular uptake [32,33].

**Figure 3:**
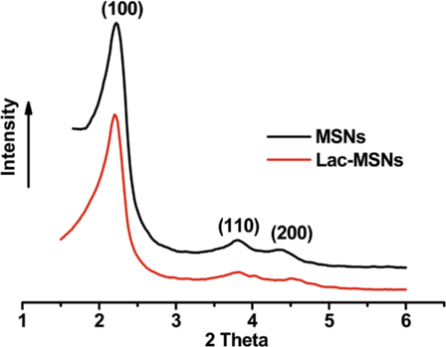
**Small-angle XRD patterns of MSNs and Lac-MSNs.**

**Figure 4:**
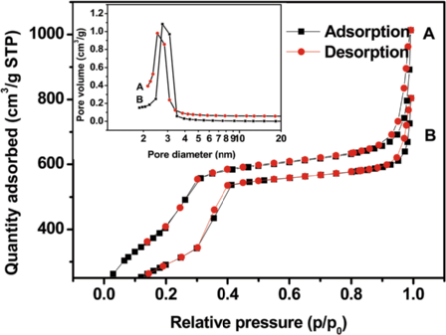
**Nitrogen adsorption/desorption isotherms of Lac-MSNs (A) and MSNs (B), with the corresponding pore size distribution shown in the insert.**

The mesostructure ordering of nanoparticles was analyzed by X-ray diffraction (XRD) patterns (Figure 3), where three distinct diffraction peaks indexed at (100), (110), and (200) revealed that both MSNs and Lac-MSNs had a highly ordered 2D hexagonal (*P*6mm) symmetry [34]. Nitrogen adsorption-desorption measurements are usually employed to obtain precise information about the structure of porous materials. As shown in Figure 4, both MSNs and Lac-MSNs exhibited the classical type-IV isotherms with H1-type hysteresis. According to the International Union of Pure and Applied Chemistry (IUPAC) classification [35], this suggests that both MSNs and Lac-MSNs have uniform mesoporous channels and relatively narrow pore size distribution (the insert of Figure 4), in consistence with the TEM images and the results of XRD. Moreover, the mean surface area, pore volume, and pore size of MSNs and Lac-MSNs were calculated as 1335 and 1012 m^2^/g, 1.85 and 1.33 cm^3^/g, 4.1 and 3.7 nm, respectively.

**Figure 5:**
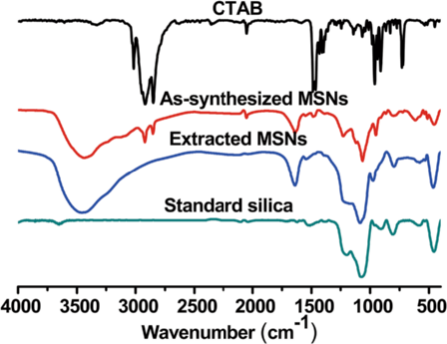
**FTIR spectra of CTAB, as-synthesized MSNs, extracted MSNs, and standard silica.**

The cationic surfactant cetyltrimethyl ammonium bromide (CTAB) was used as a mesoporous template for synthesis of MSNs and removed through postsynthesis extraction with acidic ethanol. Because the marked cytotoxicity of CTAB was reported by other researchers [36], fourier-transform infrared spectra (FTIR) analysis was carried out to confirm the complete removal of CTAB. Typically, CTAB shows two intense peaks at 2800–3200 cm^−1^, which correspond to the symmetric (2849 cm^−1^) and asymmetric (2918 cm^−1^) stretching vibrations of the methylene chains (Figure 5). These peaks were observed in as-synthesized MSNs but absent in the extracted MSNs, indicating the complete removal of CTAB through extraction. Moreover, in FTIR spectra the standard silica, as-synthesized MSNs, and extracted MSNs all showed the same characteristic peaks in the region of 400–1800 cm^−1^, indicating the MSNs had the same chemical constituents as the pure silica.

Lactose was conjugated to MSNs through the formation of Schiff base between the aldehyde group on the ring-open form of glucose moiety in lactose and the amino-silane groups in MSNs [28]. Lactose content of 2.11 μg/mg in MSNs was measured by the phenol/sulfuric acid method, indicating an efficient lactose-binding on MSNs was achieved.

### Targeting efficiency of Lac-MSNs

**Figure 6:**
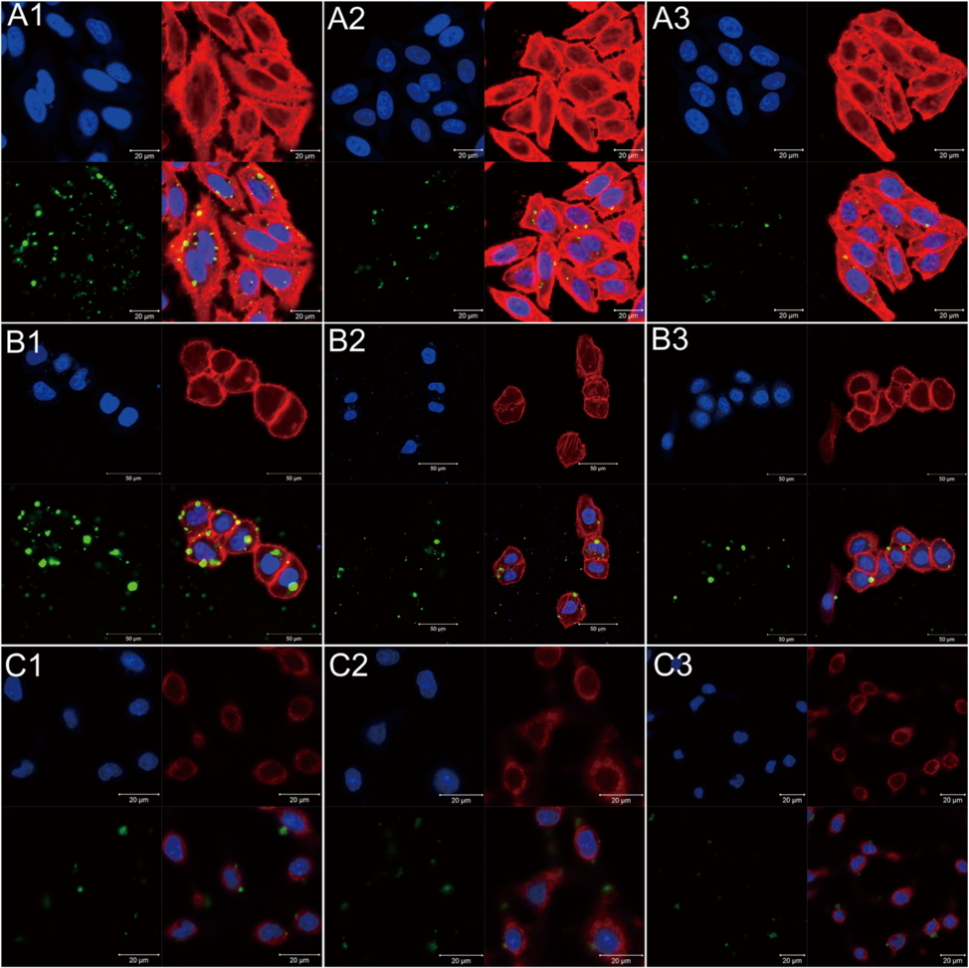
**Confocal microscopy images of different cells.** ASGPR-positive cells HepG2 **(A)** and SMMC7721 **(B)**, and ASGPR-negative cells NIH 3T3 **(C)** incubated with Lac-MSNs (1), MSNs (2), and excess free lactose with Lac-MSNs (3) for 4 h at 37°C. Cell nuclei were stained blue with DAPI, filamentous actin cytoskeletons were stained red with rhodamine phalloidin, and FITC was shown as green fluorescence.

The cellular uptake of FITC labeled nanoparticles was studied on two kinds of ASGPR-positive hepatoma cells HepG2 and SMMC7721, as well as ASGPR-negative fibroblast cells NIH 3T3 via laser scanning confocal microscope (Figure 6). The HepG2 and SMMC7721 cells incubated with Lac-MSNs showed stronger green appearance than those with MSNs, indicating that lactose modification significantly enhanced the cell uptake by ASGPR-positive cells. However, low cellular uptake by NIH 3T3 cells was observed for both Lac-MSNs and MSNs, suggesting the low affinity between ASGPR-negative cells and nanoparticles. Moreover, the cellular internalization of Lac-MSNs by HepG2 and SMMC7721 cells markedly decreased in the presence of excess free lactose. This corroborates that ASGPR on the membrane of hepatoma cells facilitates the recognition of lactose on Lac-MSNs and increases the cellular uptake through ASGPR-mediated endocytosis.

**Figure 7:**
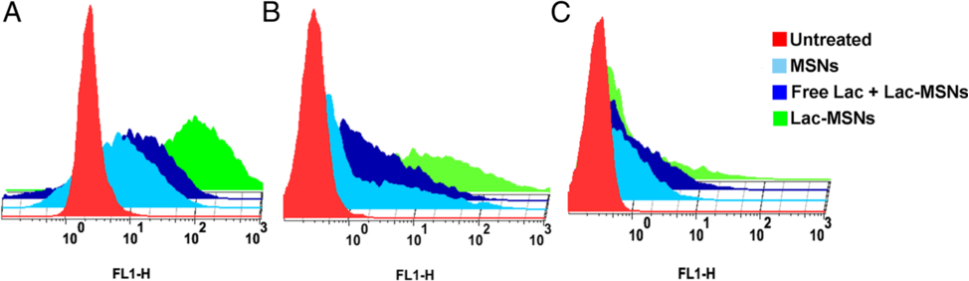
**Flow cytometry study.** ASGPR-positive cells HepG2 **(B)** and SMMC7721 **(C)**, and ASGPR-negative cells NIH 3T3 **(A)** incubated with blank medium (control), Lac-MSNs, MSNs, and excess free lactose with Lac-MSNs for 4 h at 37°C. Data represent mean ± SD (*n* = 3).

Flow cytometry was employed to quantitatively evaluate the cellular internalization of nanoparticles. The logarithmic autofluorescence intensity of untreated cells was set between 10^0^ and 10^1^, and any higher fluorescence intensity might indicate the cellular internalization of FITC labeled nanoparticles [2], as the extracellular fluorescence was already quenched by trypan blue solution [36]. As shown in Figure 7, the uptake efficiency for Lac-MSNs was 2.1 and 1.8 times higher than that for MSNs in HepG2 and SMMC7721 cells respectively. Moreover, the presence of excess free lactose markedly decreased the cellular internalization of Lac-MSNs approximately by 30% and 40% in HepG2 and SMMC7721 cells, respectively. This further proved that Lac-MSNs were transported into ASGPR-positive cells via receptor-mediated endocytosis. Consistent with the confocal microscope images, both MSNs and Lac-MSNs showed similar lower cellular uptake efficiency in ASGPR-negative NIH 3T3 cells.

Therefore, our hypothesis that lactosaminated MSNs might have a greater ability to actively target the hepatoma cells through ASGPR expressed on the cell surface was proved, and the Lac-MSNs could serve as nanoreservoirs for targeted drug delivery.

### Mechanism for nanoparticle uptake

**Figure 8:**
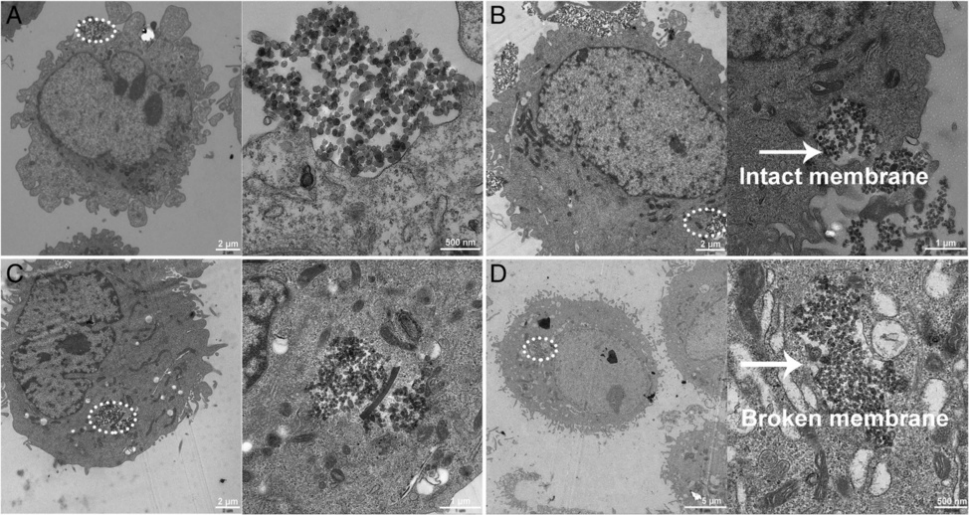
**Bio-TEM images of HepG2 cells.** HepG2 cells treated with Lac-MSNs at 37°C for 10 min **(A)**, 1 h **(B)**, 4 h **(C)**, and 24 h **(D)**. Images on the right represent the circled domains on the left with a higher resolution.

Bio-TEM observation on the ultrathin sections of HepG2 cells after being treated with Lac-MSNs for varying time was used to explore the process of cellular uptake and intracellular trafficking. Firstly, a part of Lac-MSNs were found near the cell membrane, interacting with the cell surface and inducing the cell membrane invagination after 10 min of treatment (Figure 8A). Then the cell membrane pinched off to form endocytic vesicles which carried the nanoparticles into the cytoplasm at 1 h (Figure 8B). After uptake by cancer cells via endocytosis at 4 h, the nanoparticles were processed in endosomes, as clearly marked by the circle in Figure 8C. The membrane of endosome surrounding the clumpy nanoparticles finally broke to release the nanoparticles at 24 h (Figure 8D) [37]. This step is very important, because the drug can only be released into cytoplasm after the delivery vesicle escapes from the endosome. Moreover, a large number of MSNs found in the cytoplasm maintained their spherical morphology, and no nanocarriers were found in the nucleus at 24 h, which is consistent with the literature [38,39].

**Figure 9:**
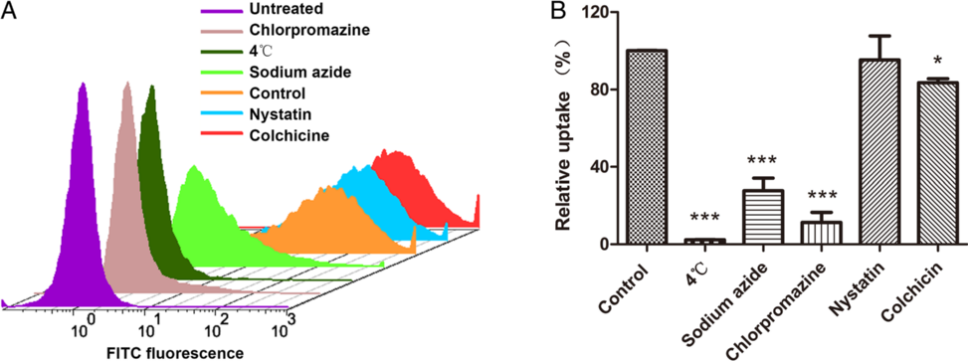
**Cellular uptake.** Flow cytometry images **(A)** and quantitative analysis **(B)** showing the cellular uptake of Lac-MSNs in the presence of different endocytic inhibitors. Data represent mean ± SD (*n* = 3). Note: ***p < 0.001 *vs* control (absence of inhibitor), *p < 0.05 *vs* control.

The influence of incubation temperature on the cellular uptake of Lac-MSNs was also studied. As shown in Figure 9, incubation of cells with Lac-MSNs at 4°C significantly impeded the uptake, resulting in approximately 90% less uptake than that of control incubated at 37°C. This indicates that the cellular uptake of Lac-MSNs requires an appropriate temperature, and endocytosis is an energy-dependent process rather than a passive diffusion.

Furthermore, a series of inhibition experiments were conducted on HepG2 cells to explore the role of specific endocytotic pathways involving in the cellular internalization. The influence of various pharmacological inhibitors on the cellular uptake of Lac-MSNs was also investigated. Sodium azide, which is widely used as an inhibitor of cellular oxidative respiration, acts by inhibiting cytochrome C oxidase and thereby blocking the cellular adenosine triphosphate (ATP) synthesis [40]. Chlorpromazine is used to inhibit the clathrin-mediated endocytosis by inhibiting the formation of clathrin vesicles. Nystatin binds sterols and disrupts the formation of caveolae, leading to inhibition of caveolae-mediated endocytosisn [41]. Colchicine is an inhibitor of non-clathrin non-caveolae-dependent endocytosis. As shown in Figure 9, the presence of sodium azide significantly decreased the cellular uptake of Lac-MSNs approximately by 70%, indicating that the uptake is energy-dependent. Compared with the uptake at 4°C, the cellular uptake with the presence of sodium azide was apparently higher, which is probably because of the presence of exogenous ATP and glucose in the media [42]. Similarly, the presence of chlorpromazine and colchicine decreased the cellular uptake of Lac-MSNs approximately by 80% and 20% respectively. In contrast, HepG2 cells pretreated with nystatin showed negligible reduction in uptake. Therefore, the results suggested that endocytosis of Lac-MSNs into HepG2 cells was an energy-dependent process predominated by clathrin-mediated endocytosis, and non-clathrin non-caveolae-dependent endocytosis may represent an additional endocytotic route. This is consistent with the reported literature [41-43], in which the endocytosis mechanism of MSNs by A549, KB, and 3T3 cells was investigated.

### Drug loading and *in vitro* release of DTX

**Figure 10:**
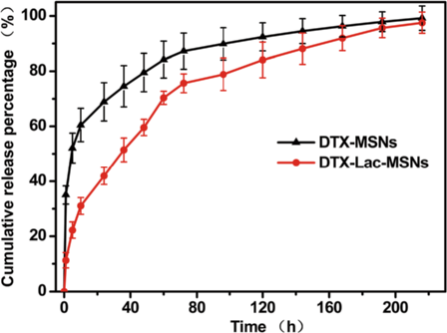
***In vitro*** **Drug release.** Release profiles of DTX from DTX-MSNs and DTX-Lac-MSNs in PBS at 37°C. Data represent mean ± SD (*n* = 3).

One critical challenge for cancer therapy is the limited availability of effective carriers for most hydrophobic anticancer drugs. In this study, hydrophobic anticancer drug DTX was successfully loaded into the channels of MSNs, obtaining drug loading of 10.1 and 12.4 nmol in 1 mg of Lac-MSNs and MSNs respectively as determined by high performance liquid chromatography (HPLC). The cumulative DTX release profiles from DTX-MSNs and DTX-Lac-MSNs in phosphate buffered saline (PBS) at 37°C are shown in Figure 10. Only 20% of DTX was released from DTX-Lac-MSNs at 10 h, showing a slower release rate as compared with DTX-MSNs. Moreover, it took 96 h for DTX-Lac-MSNs to release 80% of drug, whereas DTX-MSNs only needed half of the time. It has been reported that modifying the surface of mesoporous silica materials could restrict water diffusing into the matrix and subsequently slow down the release process [44]. So, the reduced drug release rate noted for DTX-Lac-MSNs can be explained by surface modification.

### *In vitro* cytotoxicity of DTX loaded nanoparticles

**Figure 11:**
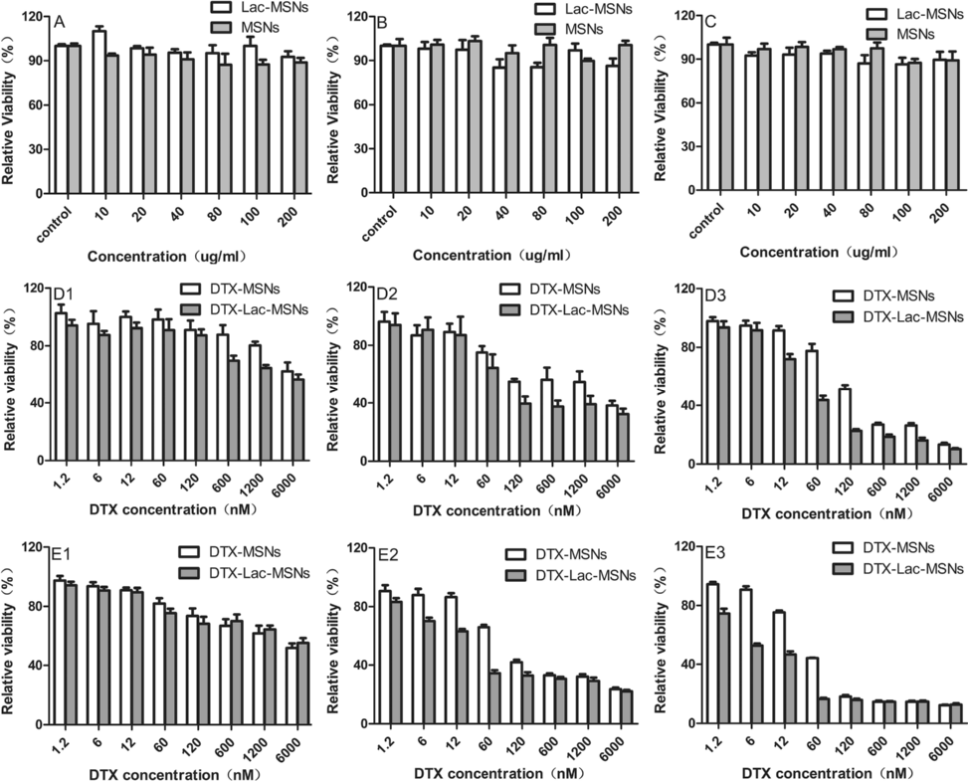
***In vitro*** **cytotoxicity analysis.** Viabilities of HepG2 **(A)**, SMMC7721 **(B)**, and NIH 3T3 **(C)** cells incubated with MSNs and Lac-MSNs for 72 h, and the viabilities of HepG2 **(D)** and SMMC7721 **(E)** cells treated with varying concentrations of DTX contained in MSNs and Lac-MSNs for 24 h (1), 48 h (2), and 72 h (3), respectively, at 37°C. Data represent mean ± SD (*n* = 6).

The biosafety of the drug carriers must be taken into consideration before application. Herein, MSNs and Lac-MSNs were incubated with HepG2, SMMC7721, and NIH 3T3 cells for 72 h at a broad concentration range of 10–200 μg/ml and their cytotoxicity was evaluated via MTT assay. As shown in Figure 11, both MSNs and Lac-MSNs showed negligible cytotoxicity in spite of sample concentration and cell species, as the cell viabilities all remained above 90%.

The *in vitro* cytotoxicity of DTX-Lac-MSNs and DTX-MSNs against both HepG2 and SMMC7721 cells was measured to assess their specific tumor targeting effect. It was found the cytotoxicity of DTX-Lac-MSNs and DTX-MSNs was strongly dependent on the drug concentration and treatment time. After 48 and 72 h treatment, both HepG2 and SMMC7721 cells exhibited appreciable level of cell death. Further calculation was performed to determine concentrations needed to cause 50% inhibition (IC50). As shown in Table 1, the IC50 values were much lower for DTX-Lac-MSNs than for DTX-MSNs against two kinds of human hepatoma cell lines (*p* < 0.05). These results suggested that Lac-MSNs transported DTX into hepatoma cells more effectively than MSNs, resulting in distinctly enhanced cytotoxicity, which was correlated to the aforementioned enhanced cellular uptake. Therefore, Lac-MSNs may potentially be used as vehicles for loading anticancer drugs and targeted cancer therapy.

**Table 1:** ***In vitro*** **cytotoxicity of drug loaded nanoparticles**

**Cells**	**Treatment**	**IC50 (nM)**	
	**Time (h)**	**DTX-MSNs**	**DTX-Lac-MSNs**
HepG2	24	---	---
	48	122.43 ± 8.02	51.6 ± 5.51^*^
	72	117.04 ± 20.38	31.55 ± 6.35^*^
SMMC7721	24	---	---
	48	72.77 ± 11.14	16.65 ± 3.31^*^
	72	32.61 ± 6.24	10.51 ± 1.01^*^

IC50 values of DTX-MSNs and DTX-Lac-MSNs against HepG2 and SMMC7721 cells after 24, 48 and 72 h of treatment. Data represent mean ± SD (*n* = 6).

*Significantly different from DTX-MSNs according to a Student’s *t*-test and Mann–Whitney *U* test (*p* < 0.05).

## Conclusions

In summary, a hepatoma targeting drug delivery system was successfully constructed by conjugation of mesoporous silica nanoparticles with the active targeting agent lactose. The Lac-MSNs were demonstrated to specifically target ASGPR-positive HepG2 and SMMC7721 cells, and their internalization into hepatoma cells is an energy-consuming process and predominated by clathrin-mediated endocytosis. Water insoluble anticancer drug DTX was effectively loaded in the pores of Lac-MSNs, showing significantly enhanced cytotoxicity. Therefore, Lac-MSNs provide a promising approach for targeted intracellular anti-cancer drug delivery.

## Materials and methods

### Materials

Docetaxel (DTX, purity > 99%) was purchased from Yikangsida Med. Tech. Ltd. (Beijing, China). Lactose monohydrate and sodium cyanoborohydride were obtained from Aladdin (Shanghai, China). Cetyltrimethyl ammonium bromide (CTAB), tetraethoxysilane (TEOS), (3-aminopropyl) triethoxysilane (APTES), 3-(4,5-dimethylthiazol-2-yl)-2,5-diphenyltetrazolium bromide (MTT), 4',6-diamidino-2-phenylindole (DAPI), phalloidin-tetramethylrhodamine B isothiocyanate conjugate (rhodamine phalloidin), and fluorescein isothiocyanate (FITC) were purchased from Sigma-Aldrich (St Louis, MO, USA). Propidium iodide (PI) and Hoechst 33258 were obtained from MP Biomed. (Santa Ana, USA). Chlorpromazine, nystatin, colchicines, and sodium azide were obtained from Yuelai Med. Tech. Ltd. (Xi’an, China). Dulbecco’s modified Eagle’s medium (DMEM), trypsin-EDTA, fetal bovine serum (FBS), and penicillin-streptomycin were acquired from GIBCO (Gaithersburg, MD, USA). All other reagents used were of analytical grade.

Human hepatoma cell lines HepG2 and SMMC7721, and mouse embryonic fibroblast cell line NIH 3T3 were obtained from Shanghai Institute of Cell Biology (Chinese Academy of Sciences, Shanghai, China). The cells were cultured in Dulbecco’s modified eagle’s medium (DMEM) supplemented with 10% fetal bovine serum and 100 U/mL of penicillin-streptomycin, and maintained at 37°C in a humidified incubator containing 5% CO_2_. The media were changed every 2 days prior to experimental operation.

### Methods

#### Synthesis of MSNs

Mesoporous silica nanoparticles were synthesized in alkaline media using CTAB as the template and TEOS as silicon source according to Zink’s report with minor modification [45]. Briefly, 11 mg of FITC was dissolved in 6 mL of absolute ethanol and then 24 μL of 3-aminopropyl triethoxysilane (APTES) was added in. The solution reacted in the dark for 2 h and then 5 mL of TEOS was added. In another three-necked flask, 1.0 g of CTAB and 0.28 g of sodium hydroxide were dissolved in 480 mL of water, and the resulting mixture was constantly stirred at 80°C till CTAB was completely dissolved and the temperature became stable. Subsequently, the mixture of TEOS and FITC-APTES was added dropwise in the flask, and 2 h later, the particles were collected by centrifugation, washed with water till the filtrate was neutral, and rinsed twice with alcohol before dried at 60°C.

#### Synthesis of lactosaminated MSNs (Lac-MSNs)

Briefly, 1 g of MSNs were dispersed in 150 mL of toluene, followed by adding 2 mL of NH_2_-silane (APTES) and reaction for 4 h at 120°C. Then the obtained particles were centrifuged, washed with absolute ethanol, and dried at 60°C. Subsequently, 500 mg of the dried nanoparticles were mixed with 6 mL of lactose solution (0.34 g/L) and 6 mL of sodium cyanoborohydride solution (0.31 g/L) sequentially. With gentle shaking several times daily, the mixture was allowed to react for 7 days. Finally, the Lac-MSNs were centrifuged, washed with water to remove the unconjugated lactose, and dried at 60°C. To further remove the template, 500 mg of dry nanoparticles were redispersed in 100 mL of absolute ethanol containing 2 mL of concentrated hydrochloric acid and refluxed for 24 h. Then the particles were collected and washed to remove the template.

### Characterization of MSNs

The morphology of MSNs was characterized by SEM (JSM-6330 F, JEOL, Japan). The samples were sputter-coated with gold for two cycles prior to imaging. Mesostructure of the nanoparticles was observed by TEM (JEM-1400, JEOL, Japan) with a drop of dispersed sample solution being deposited on a carbon-coated copper grid and dried at room temperature before examination. The particle size of MSNs was measured at 25°C by dynamic light scattering at a scattering angle of 90° using Zetasizer Nano ZS90 (Malvern Instruments, Worcestershire, UK). The mesostructure ordering was analyzed by small-angle X-ray diffraction (SAXRD, D/MAX 2200VPC, Tokyo, Japan) using Cu Kα radiation with 2θ in the range of 0.6°-6° at a scanning rate of 0.5°/min. Brunauer-Emmett-Teller (BET) surface area, pore volume and diameter distribution of MSNs were measured at −196°C by using a surface area and pore size analyzer (ASAP 2020C, Micromeritics, USA). FTIR spectra of MSNs were obtained by using a FTIR spectrophotometer (Bruker, German) to scan over a region of 400–4000 cm^−1^ on a thin KBr slice containing MSNs.

### Lactose content in Lac-MSNs

Lactose content was measured by the phenol/sulfuric acid method [46]. Since the secondary amine formed between amino-MSNs and glucosyl by reductive amination is acid-stable, only galactose is produced during the hydrolysis [47]. Briefly, 2 mL of standard galactose solution at various concentrations, 1 mL of phenol (5%), and 4 mL of concentrated sulfuric acid were added in a tube. The tube was sealed and allowed to react for 15 min, and the absorbance after reaction was measured at 490 nm to build a standard curve. Then, dispersion of Lac-MSNs (50 mg) in deionized water (2 mL) was treated similarly, the absorbance was measured and the lactose content was calculated according to the standard curve.

### Confocal microscopy study

The cellular uptake of nanoparticles was visualized by confocal microscopy [36]. HepG2, SMMC7721, and NIH 3T3 cells were seeded at 1 × 10^5^ per dish in special glass dishes and allowed to attach for 24 h. Then, Lac-MSNs and MSNs suspensions in DMEM at a final concentration of 50 μg/mL were added in. After 4 h of incubation, the medium was removed and the cells were washed with cold PBS (pH 7.4) three times. Trypan blue PBS solution (0.4%) was added to quench any fluorescence outside the cells for 10 min. Afterwards, the cells were fixed with 4% paraformaldehyde at room temperature for 10 min and extracted with 0.1% Triton X-100 in PBS for 3 min. Subsequently, the filamentous actin cytoskeleton was stained with 200 ng/mL rhodamine phalloidin for 20 min, followed the nuclei staining with DAPI for 10 min. Finally, the samples were analyzed with the laser scanning confocal microscope (LSCM, Zessi LSM 710, Germany).

### Flow cytometry study

Cellular uptake was quantitatively analyzed by flow cytometry. HepG2, SMMC7721, and NIH 3T3 cells were seeded into 12-well plates at the density of 2 × 10^5^ cells per well and allowed to attach for 24 h. Then, Lac-MSNs and MSNs suspensions in DMEM at a final concentration of 50 μg/mL were added in for cell incubation. After 4 h of incubation, the medium was removed and 0.4% trypan blue PBS solution was added to neutralize the extracellular fluorescence. Then the cells were harvested by trypsinization, collected by centrifugation, and resuspended in 4% paraformaldehyde PBS solution. Finally, the collected cells were analyzed by a Beckman Coulter EPICS XL flow cytometer (Beckman Coulter, Fullerton, CA, USA). The regular DMEM was applied as the blank control. All the tests were performed in triplicate.

In order to assess the competitive uptake efficacy, the cells were preincubated with 50 μg/mL of excessive free lactose for 30 min at 37°C. Then 50 μg/mL of Lac-MSNs were added for incubation at 37°C for another 4 h. Following the similar procedures above, the fluorescent images were taken with confocal microscopy and the quantitative results were measured by flow cytometer.

### Cell transmission electron microscopy (TEM)

Bio-TEM observation was performed on ultrathin sections of HepG2 cells after being treated with Lac-MSNs to reveal the endocytic process of the nanoparticles into cancer cells and their intracellular locations [48]. Cells were seeded into 12-well plates at the density of 2 × 10^5^ cells per well and allowed to attach for 24 h. Then Lac-MSNs were added in for incubation at 37°C for 10 min, 1 h, 4 h, and 24 h. After that, the cells were harvested and centrifuged at 4000 rpm for 1 min, immediately fixed with 2.5% glutaraldehyde solution in PBS for at least 1 h, post-fixed with 1% aqueous osmium tetroxide for another hour, dehydrated by ethanol series, washed three times with acetone, and embedded in Spurr resin medium overnight. Ultrathin sections of the cells were obtained by 300 mesh copper grids and contrasted with 0.3% lead citrate and 50% uranyl acetate. Finally, the samples were visualized with TEM.

### Endocytosis-inhibition experiments

A series of endocytosis-inhibition experiments were performed on HepG2 cells to further investigate the endocytosis mechanism of Lac-MSNs as follows [41,42]. Cells were cultured in a 12-well plate at the density of 2 × 10^5^ cells per well for 24 h. First, the cells were pretreated with various endocytosis inhibitors for 30 min, including chlorpromazine (20 μg/mL) for inhibition of clathrin-mediated endocytosis, nystatin (30 μg/mL) for inhibition of caveolae-mediated endocytosis, colchicine (20 μg/mL) for inhibition of non-clathrin non-caveolae-dependent endocytosis, and sodium azide (3 mg/mL) for ATP. Then the medium was replaced with 50 μg/mL Lac-MSNs suspension. After incubation at 37°C for 4 h, the cells were harvested and analyzed by flow cytometer. Another uptake study was performed similarly at 4°C for inhibition of cell respiration to further determine whether the uptake of Lac-MSNs into human hepatoma cells was energy-dependent. All the tests were performed in triplicate.

### Drug loading and release study

Model drug DTX was loaded in the pores of the nanoparticles by wetness impregnation method [21]. MSNs and Lac-MSNs (100 mg each) were added to 5 mL of ethanol solution containing 5 mg/mL of DTX, respectively. Magnetic stirring was applied at room temperature for 24 h to maximize drug loading in the pores. Then the drug-loaded nanoparticles (DTX-MSNs and DTX-Lac-MSNs) were collected by centrifugation, washed twice with PBS to remove the free drug on the particle surface, and dried under vacuum.

The drug-loaded nanoparticles (10 mg) were resuspended in methanol because of the high solubility of DTX in methanol to determine the amount of drug actually loaded in the nanoparticles. The suspension was sonicated to dissolve DTX from the pores, and then the supernatant was collected by centrifugation. This process was repeated twice to ensure the loaded drug was completely removed from the pores. The concentration of DTX in the supernatant was determined by HPLC (Daojing, Japan).

Pretreated dialysis bags with dialyzer molecular weight cutoff 14,000 Da were used in the drug release experiments. DTX-MSNs and DTX-Lac-MSNs samples (20 mg each) were dispersed in 2 mL of PBS, and the solutions were placed into the pretreated dialysis bags. The sealed bags were immersed in 10 mL of PBS and shaken at 100 rpm at 37°C. The release medium was taken and replaced with fresh medium at given time intervals. Each release study was performed in triplicate. The concentration of DTX in samples was measured by HPLC.

### Cytotoxicity study

The cytotoxicity of MSNs, Lac-MSNs, DTX, DTX-MSNs, and DTX-Lac-MSNs was evaluated against HepG2 and SMMC7721 cells by MTT viability assay. Cells were seeded in 96-well plates at a density of 5 × 10^3^ cells per well. After incubation in 5% CO_2_ at 37°C for 24 h, the medium was replaced with 200 μL of fresh medium containing different concentrations of samples. Cells treated with pure medium were used as the blank control. After incubation with the samples for 24, 48, and 72 h, the medium was replaced with 20 μL of MTT (5 mg/mL) and 180 μL of fresh medium for another 4 h of incubation at 37°C. Finally, 150 μL of DMSO was added in each cell to dissolve the purple formazan crystals and the absorbance was measured at 490 nm by an ELX 800 micro-plate reader. The cytotoxicity was calculated as the percentage of cell viability as compared with the blank control, and data were expressed as mean ± standard deviation (SD) of six independent wells. The IC50 values of different formulations were calculated via nonlinear regression of the log(dose)-response profiles using GraphPad Prism 5.

### Statistical analysis

IC50 values of DTX-MSNs and DTX-Lac-MSNs were compared using the Student’s *t*-test and Mann–Whitney *U* test following normality and equal variance tests (SPSS 13.0). Statistical analysis of the effects of various pharmacological inhibitors on the cellular uptake of Lac-MSNs was performed using a one-way ANOVA (SPSS 13.0). The post-hoc comparisons of the means of individual groups were performed using least significant difference test. Differences were considered significant if *P* < 0.05.

## Abbreviations

MSNs: Mesoporous silica nanoparticles
Lac: Lactose
Lac-MSNs: Lactosaminated mesoporous silica nanoparticles
ASGPR: Asialoglycoprotein receptor
DTX: Docetaxel
CTAB: Cetyltrimethyl ammonium bromide
TEOS: Tetraethoxysilane
APTES: 3-Aminopropyl triethoxysilane
DAPI: 4',6-diamidino-2-phenylindole
FITC: Fluorescein isothiocyanate
SEM: Scanning electron microscopy
TEM: Transmission electron microscopy
XRD: X-ray diffraction
MTT: 3-(4,5-dimethylthiazol-2-yl)-2,5-diphenyltetrazolium bromide
DLS: dynamic light scattering
FTIR: Fourier-transform infrared spectra
