# Erratum to: Lactosaminated mesoporous silica nanoparticles for asialoglycoprotein receptor targeted anticancer drug delivery

**DOI:** 10.1186/s12951-015-0104-6

**Published:** 2015-11-30

**Authors:** Guilan Quan, Xin Pan, Zhouhua Wang, Qiaoli Wu, Ge Li, Linghui Dian, Bao Chen, Chuanbin Wu

**Affiliations:** School of Pharmaceutical Sciences, Sun Yat-Sen University, Guangzhou, 510006 People’s Republic of China; Guangzhou Neworld Pharmaceutical Ltd. Co., Guangzhou, 510006 People’s Republic of China; School of Pharmaceutical Sciences, Guangdong Medical College, Dongguan, 523808 People’s Republic of China

## Erratum to: J Nanobiotechnol DOI 10.1186/s12951-015-0068-6

Panel A, B, and C from Figure 7 (Fig. 1 here) of this work [1] was generated using HepG2, SMMC7721, and NIH 3T3 cells, respectively. After publication of this work, we noted that they were inadvertently labelled as NIH 3T3, HepG2 and SMMC7721 cells. The figure caption of Figure 7 has now been corrected in this erratum.

**Figure 1:**
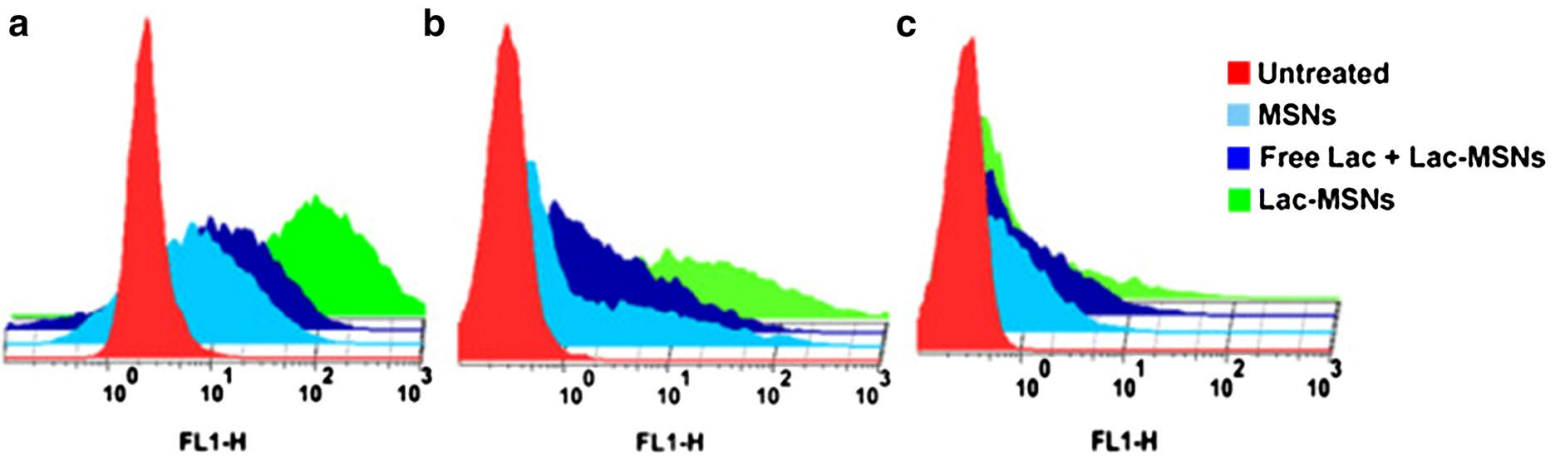
** Flow cytometry study.** ASGPR-positive cells HepG2 (**A**) and SMMC7721 (**B**), and ASGPR-negative cells NIH 3T3 (**C**) incubated with blank medium (control), Lac-MSNs, MSNs, and excess free lactose with Lac-MSNs for 4 h at 37°C. Data represent mean ± SD (*n* = 3).
